# Spatially Resolved Differentiation of Functional Degradation and Perforating Structural Defects in Membrane Electrode Assemblies Using Diffusion-Cell Coupled DC-SECM

**DOI:** 10.1021/acsmeasuresciau.5c00071

**Published:** 2025-08-12

**Authors:** Susanne Thiel, Maik Eichelbaum

**Affiliations:** Institute for Applied Hydrogen Research, Electro- and Thermochemical Energy Systems (H2Ohm), 38920 Technische Hochschule Nürnberg Georg Simon Ohm, Prinzregentenufer 47, 90489 Nuremberg, Germany

**Keywords:** scanning electrochemical microscopy, polymer electrolyte membrane, catalyst-coated membrane, diffusion cell, degradation

## Abstract

In order to increase the lifetime of polymer electrolyte membrane (PEM) fuel cells (PEMFCs) and water electrolyzers (PEMWEs), understanding local degeneration processes in membrane electrode assemblies (MEAs) is crucial. By a combination of scanning electrochemical microscopy (SECM) with a flow-through diffusion cell (DiffC-DC-SECM) and ferrocyanide and protons as redox mediators, a spatially resolved analytical method was developed that can differentiate between different functional and structural degeneration phenomena in the aging process of a membrane. An SECM scan at cathodic potential detects the diffusion of protons through the membrane and thus its through-plane proton conductivity, while a second SECM scan at anodic potential visualizes the diffusion of the iron complex through the membrane, thus perforating structural damage such as cracks and holes. The method was successfully validated for the spatially resolved differentiation of membrane damage in pristine PEMs and catalyst-coated membranes (CCMs) with artificial holes, chemically aged CCMs, and MEAs in fully assembled operational PEMFCs aged by an open-circuit voltage membrane accelerated stress test. DiffC-DC-SECM thus provides a powerful technique with high local resolution for membrane integrity testing under realistic operation conditions to develop long-term durable materials for PEMFCs and PEMWEs.

## Graphical abstract

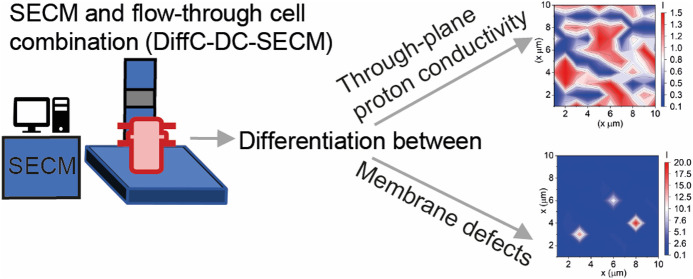

## 1 Introduction

In 2022, the transport sector was the largest source of greenhouse gas emissions in the European Union (EU), accounting for about 26% of total emissions.^1^ Achieving the EU’s 2050 net-zero greenhouse gas emissions target requires a quick shift toward low-emission alternatives. Hydrogen-powered driving systems, particularly based on polymer electrolyte membrane fuel cells (PEMFCs), are promising due to their long-range and short refueling times, especially for heavy-duty vehicles.^2–6^ To enable their widespread use in this sector, PEMFCs must meet high standards of efficiency and durability, with the U.S. Department of Energy (DoE) setting a target of 25,000 operating hours by 2030, equivalent to around 1,000,000 miles, and an ultimate target of 30,000 h lifetime.^4^ Similarly, polymer electrolyte membrane water electrolyzers (PEMWEs) must also demonstrate high efficiency and durability to support large-scale green hydrogen production and complement the anticipated growing hydrogen demand. PEMWEs enable the efficient splitting of water to convert surplus renewable electricity into hydrogen. As a universal energy vector, hydrogen has great potential to become the backbone of a future defossilized economy.^7,8^

A major factor limiting fuel cell and electrolyzer lifetime is the degradation of the polymer electrolyte membrane (PEM), which is highly prone to aging.^9^ The PEM usually consists of a hydrophobic poly­(tetrafluoroethylene) backbone with hydrophilic sulfonic acid side chains. It enables proton conduction, gas separation, and electrical insulation in PEMFCs and PEMWEs.^7,10^ In particular, in heavy-duty vehicle applications, enhanced chemical and mechanical stability is crucial due to higher operating temperatures and pressures and the need to reduce gas crossover for improved efficiency and durability.^11^ PEM degradation involves various decomposition mechanisms. During fuel cell operation, reactive species such as hydrogen peroxide and hydroxyl radicals are generated, which can attack the polymer structure. Hydrogen peroxide forms as a byproduct of oxygen reduction (eq 1), predominantly at the anode due to oxygen crossover and the lower potential,^12^ but may also form at the cathode when the potential is below 0.696 V.^9^ In the presence of transition metal ions such as Fe^2+^, Co^2+^, or Cu^2+^, it can further react to form highly reactive hydroxyl radicals. These ions may enter the system during manufacturing or leach from metallic components such as bipolar plates.^9,12^

1 $$O_{2}+2H^{+}+2e^{-}\to H_{2}O_{2}$$

These radical attacks can occur at different sites within the membrane, and an overview of the potential mechanisms is provided in Figure 1. The consequences of degradation depend on the site of attack. For instance, attacks on the sulfonic acid groups are often associated with a loss of these acid groups, leading to a reduction in the proton conductivity. In contrast, other forms of attack primarily result in the thinning of the membrane, which can ultimately lead to the formation of cracks and holes.^9^ In addition to radical-induced degradation, other aging mechanisms such as ionic contamination and mechanical degradation also affect the functional properties and lifetime of PEMs.^9,13–16^

**Figure 1:**
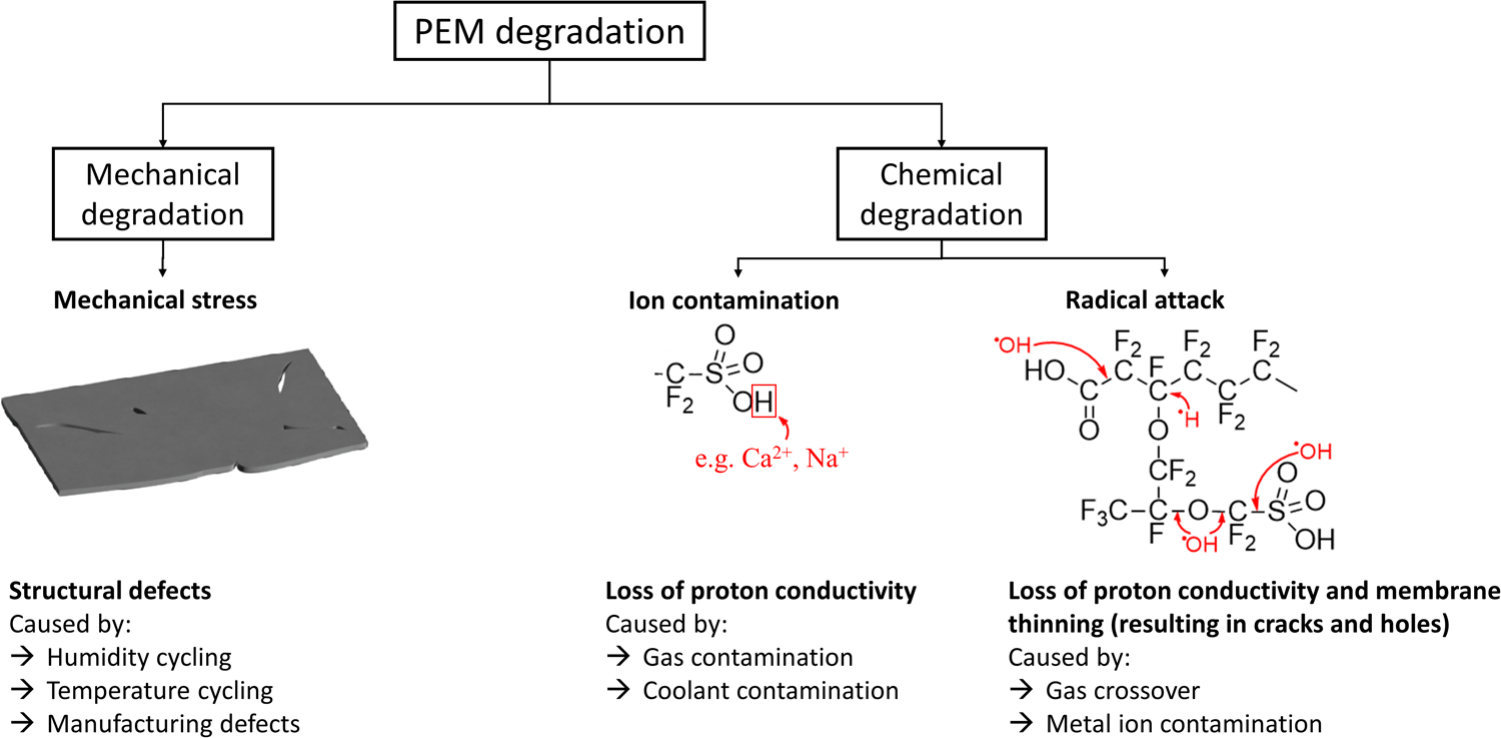
Overview of the characteristics of degradation in PEMs.

The radical-induced degradation occurring during fuel cell and electrolyzer operation can be artificially simulated by treatment of a pristine membrane with the Fenton reagent (eq 2). In this process, the reaction between iron­(II) ions and hydrogen peroxide generates radicals that can attack the polymer structure.^17,18^

$$H_{2}O_{2}+{\mathrm{Fe}}^{2+}\to {\mathrm{Fe}}^{3+}+{\mathrm{OH}}^{•}+{\mathrm{OH}}^{-}$$ 2

PEM degradation in fuel cells and electrolyzers is often not directly observable, complicating damage detection and root cause analysis. Advanced characterization methods are therefore essential to understand degradation mechanisms and ensure the long-term stability of both conventional and novel PEM materials. A variety of analytical methods have been used to study PEMs in fuel cells, each focusing on different membrane properties. Ex situ techniques such as ion exchange capacity (IEC) and Fourier transform infrared spectroscopy (FTIR) analyze isolated PEMs to determine chemical composition and the integrity and number of proton-conducting sulfonic acid groups.^19,20^ Electrochemical methods, including electrochemical impedance spectroscopy (EIS) and linear sweep voltammetry (LSV), can provide information on proton conductivity and hydrogen permeability. Increased hydrogen permeability usually indicates membrane thinning or the presence of perforative structural defects.^21–23^ Ion chromatography enables quantification of fluoride ions in effluent water, serving as an indirect indicator of membrane degradation.^23^ For spatially resolved analysis, ex situ methods such as X-ray computer tomography (XCT) and scanning electron microscopy (SEM) have been employed to visualize morphological changes like cracks, holes, or thinning.^21–25^ For CCM characterization, SEM typically requires destructive cross-sectional preparation, limiting the analysis to small areas. Furthermore, neither SEM nor XCT yields direct data on physicochemical functional properties such as proton conductivity.

Due to its high spatial resolution and versatile applicability, scanning electrochemical microscopy (SECM) holds great potential for the characterization of components in electrochemical cells. Depending on the specific application, the measurement can be optimized either for scanning large areas or for the highest possible lateral resolution. SECM enables the investigation of localized phenomena at the micro- to nanometer scale, thereby providing valuable insights into electrochemical properties. Recently, we applied intermittent-contact alternating-current SECM (IC-AC-SECM) and a diffusion-cell coupled direct-current SECM for the characterization of defects in PEMs.^26,27^ In our current study, we demonstrate that an advanced flow-through diffusion-cell coupled DC-SECM technique (DiffC-DC-SECM) enables the spatially resolved differentiation of functional degradation, i.e., changes in the through-plane proton conductivity, and of perforative structural defects, such as cracks and holes, in CCMs and membrane electrode assemblies (MEAs) of fully assembled and operational PEMFCs. The method was validated for the spatially resolved differentiation of membrane damage in pristine PEMs and CCMs with artificial holes, chemically aged CCMs, and MEAs in PEMFCs aged by a standardized open-circuit voltage (OCV) membrane accelerated stress test (AST) as proposed by the DoE. The results obtained using DiffC-DC-SECM were validated by comparison with established characterization methods such as IC-AC-SECM, LSV, thermography, and SEM.

## 2 Experimental Section

### 2.1 Membrane Preparation

The investigated single membrane (Nafion type N-115, 125 μm thickness, QuinTech) was initially activated by stirring in ultrapure water (Milli-Q Integral 3 system, 18.3 MΩ) at 80 °C for 30 min. The investigated CCMs (GOREⓇ PRIMEAⓇ 3-layer MEA with Pt/C catalyst layers on both sides, Type A510.1/M775.15/C586.4, 15.5 μm ePTFE membrane thickness, 25 cm^2^ active area, W.L. Gore & Associates) were used without prior activation.

The samples with artificial holes were prepared by manually piercing two holes with a needle into pristine PEM and CCM, respectively. The preparation of the sample that was artificially aged by Fenton’s reagent was carried out by immersing a pristine CCM in a solution of 16 mg L^-1^ of iron­(II) ions (iron­(II) chloride hydrate, extra pure, Carl Roth) in 30 wt % hydrogen peroxide (for analysis, Carl Roth) at 70 °C for 8 h. After 4 h, the solution was replaced with a fresh one. Finally, the CCM was rinsed with ultrapure water. The treatment of the CCM that was realistically aged in a fully assembled fuel cell is described in the next chapter. For the DiffC-DC-SECM investigation, the GDLs were carefully removed from the MEA with tweezers.

All samples were stored in ultrapure water after their respective treatments until measurement. An overview of the investigated samples and their used acronyms, with information on their preparation conditions, is shown in Table 1.

**Table 1:** Name and Description of the Investigated Samples

abbreviation	sample description
PEM-AH	pristine PEM with artificial holes
CCM-AH	pristine CCM with artificial holes
CCM-AA	CCM artificially aged with Fenton’s reagent
CCM-RA	CCM realistically aged in a fully assembled fuel cell

### 2.2 Fuel Cell Accelerated Stress Test

The chemical stability of the membrane in a fully assembled fuel cell was investigated by an AST at OCV as proposed by the DoE.^28^ All electrochemical measurements for analysis and the AST were conducted using a commercial fuel cell test system equipped with a frequency response analyzer (881), two potentiostats (850e and 885), and a 25 cm^2^ PEM fuel cell fixture (all from Scribner Associates). For the AST, a CCM (same type as described in chapter 2.1) was assembled with two GDLs, each with one-sided microporous coating (H23C8, Freudenberg) to build a complete 7-layer MEA. The MEA was placed between two graphite flow-fields with serpentine flow patterns (Scribner Associates) and inserted into the PEMFC fixture. More detailed information on the test conditions and electrochemical analysis results can be found in the Supporting Information.

### 2.3 Scanning Electrochemical Microscopy

For SECM measurements, a scan station (M470) equipped with a potentiostat (SP-300, both from BioLogic) was used. A platinum wire ultramicroelectrode (UME) with a diameter of 15 μm was used as the working electrode (WE), an Ag/AgCl electrode (3 M KCl, RE-1CP) was used as the reference electrode (RE), and a platinum sheet (all from BioLogic) was used as the counter electrode (CE).

For the characterization of the electrode, the RG ratio was determined. The procedure is described in detail in the Supporting Information (Figure S2). An RG ratio of 24.8 was obtained as a result, based on a platinum wire radius of 15.4 μm determined from the steady-state current of the cyclic voltammogram. Although the obtained RG ratio of 24.8 exceeds the typically recommended range for UMEs, the electrode was still well suited for the intended application. The relatively large RG ratio may reduce lateral resolution due to hindered radial diffusion at the glass insulation. However, the electrode enabled stable steady-state currents and showed the expected sigmoidal shape in the cyclic voltammogram, confirming its electrochemical functionality. Given the focus on micrometer-scale membrane defects, the electrode provided sufficient spatial resolution and robust signal quality to reliably detect perforations and thinned regions. For future studies aiming at the detection of nanoscale damage, smaller UMEs with optimized RG ratios would be required to achieve higher lateral resolution, although this would come at the expense of longer measurement times and reduced scan areas.

The expected diameter of holes formed within the PEM as a result of accelerated stress testing typically ranges from approximately 50–500 μm, with some reports indicating hole sizes up to 1.5 mm.^22–25^ Therefore, the selected diameter of the UME and a step size of 20 μm were chosen to achieve an optimal balance between the measurement time and the required spatial resolution.

IC-AC-SECM measurements were performed using an electrochemical cell (BioLogic) and tap water as the electrolyte, and the scan velocity was set to 100 μm s^–1^. The DC potential applied to the WE was 0 V, while the AC potential was 50 mV with an AC frequency of 100 kHz.

The use of tap water in this context is supported by the previous literature, including Biologic’s Application Note and the publication by Catarelli et al.^29,30^ In our experiments, the PEM samples were stored in ultrapure water and exposed to only tap water during short measurement periods. These were kept as brief as possible to minimize any risk of contamination. Under these carefully controlled conditions, no electrode fouling or instability was observed.

In previous DC-SECM measurements,^27^ spatial resolution was strongly limited by diffusion processes, resulting in blurred detection of defects such as holes and cracks. To improve resolution, the conventional diffusion cell was replaced by a flow-through diffusion cell (PermeGear), in which the electrolyte solutions are continuously exchanged using pumps. The outlet of the acceptor reservoir was positioned in close proximity to the working electrode by using a tube, effectively preventing the local accumulation of the redox mediator and minimizing the risk of contamination of the sample by free ions from the donor solution.

For the continuous exchange of both electrolytes, a pump flow rate of 0.11 mL min^–1^ (50 mL syringe, NE-300, Dissolution Accessories) was set at the donor inlet and a pump flow rate of 0.09 mL min^–1^ (L-6200, Merck) at the donor outlet. For the acceptor reservoir, both the inlet and outlet pump flow rates were set to 1.5 mL min^–1^ (L-6200, Merck). The lower part of the diffusion cell (donor reservoir) was filled with a solution containing 0.01 M sulfuric acid (analytical grade, Bernd Kraft), 0.1 M potassium ferrocyanide (analytical grade, Carl Roth), and 0.1 M potassium chloride (analytical grade, Carl Roth). The upper part of the diffusion cell (acceptor reservoir) was filled with a 0.1 M potassium chloride solution. Ferrocyanide was selected due to its well-defined, reversible redox behavior and its inability to permeate intact polymer electrolyte membranes.

The PEM or CCM sample to be investigated was positioned between the donor and acceptor reservoirs, and the cell was manually assembled and clamped. The electrodes for the SECM measurements were placed in the upper reservoir. For area scans, the electrode potential was set to −0.5 V vs Ag/AgCl for proton detection and +0.5 V vs Ag/AgCl for ferrocyanide detection. The scan velocity was set to 200 μm s^–1^ for both detection modes.

### 2.4 Thermographic Measurements

Thermography can be used to detect structural perforative defects, such as cracks and holes, in fuel cell MEAs. It is based on the effect that hydrogen gas, which diffuses through perforative defects in the membrane, reacts immediately after contact with air and forms a temperature hotspot at the location of the defect. Thermographic measurements of the MEA were performed both before and after the OCV AST and were run in a slightly modified fuel cell test system (Scribner Associates). For this purpose, the cathode end plate of the fuel cell fixture was replaced with a 20 mm thick AlSiMg1 counter plate featuring a square opening of 55 mm × 55 mm. The temperature distribution on the MEA was measured by using a high-performance infrared camera (PI 640i, Optris). The camera was positioned perpendicular to the surface of the MEA to ensure optimal imaging. A fuel mixture of 20% hydrogen in nitrogen was supplied to the anode by alternating hydrogen flow for 1 s and nitrogen flow for 4 s via the test stand’s gas control system, maintaining a flow rate of 0.05 L min^–1^. Both the cell heater and humidifier were deactivated, and the heating sleeve at the connection between the gas line and the cell mount was removed to minimize background temperature and optimize accuracy. No back-pressure was applied during the test.

### 2.5 Scanning Electron Microscopy (SEM)

For the SEM measurements, PEM and CCMs were dried in a muffle furnace (B410, Nabertherm) at 72 °C for 8 h. Subsequently, the samples were sputtered with gold (Q150R ES Plus, Quorum) and analyzed with benchtop SEM (5 keV, secondary electron detection, JCM-6000, JEOL). For the preparation of the cross-section from the pristine MEA, a small piece was cut and polished at −80 °C with a cross-section polisher (IB-19520 CCP, JEOL) to obtain a clean and representative cross-section. Subsequently, the sample was analyzed by field emission SEM (5 keV, backscattered electron detection, JSM-IT800, JEOL).

## 3 Results and Discussion

For the sake of clarity, a brief summary of the DiffC-DC-SECM and IC-AC-SECM measurement techniques is provided here. In AC-SECM, an alternating potential is applied to the WE, and the resulting current is measured.^31^ IC control ensures a constant electrode–sample distance, allowing separate characterization of topography and local conductivity.^29^ A key advantage of AC-SECM is that it requires no redox mediator, enabling nondestructive measurements in tap water without contaminating the sample.^29,31^ In contrast, DC-SECM measurements require a redox mediator that undergoes either oxidation or reduction at the WE, depending on the applied potential.^32^ To implement the DC-SECM technique in this study, a diffusion cell configuration was employed. The diffusion cell consists of donor and acceptor reservoirs separated by a sample (Figure 2a). Using DC-SECM, the tip current at a set potential reflects the local concentration of the redox mediator diffusing through the membrane. To assess proton conductivity, sulfuric acid is placed in the donor reservoir and a potassium chloride solution is placed in the acceptor reservoir. When a sufficient cathodic bias is applied, an increased current is measured (Figures 2b and S3a) due to proton reduction to hydrogen (positive feedback).^32,33^ The signal intensity correlates with the local through-plane proton conductivity. However, if perforating membrane defects are present, the protons diffuse unhindered, leading to increased peak currents (positive feedback), making it impossible to distinguish between high conductivity and membrane damage based on proton transport alone (Figure S3b). To address this limitation, a complementary measurement is performed using ferrocyanide as the redox mediator.

**Figure 2:**
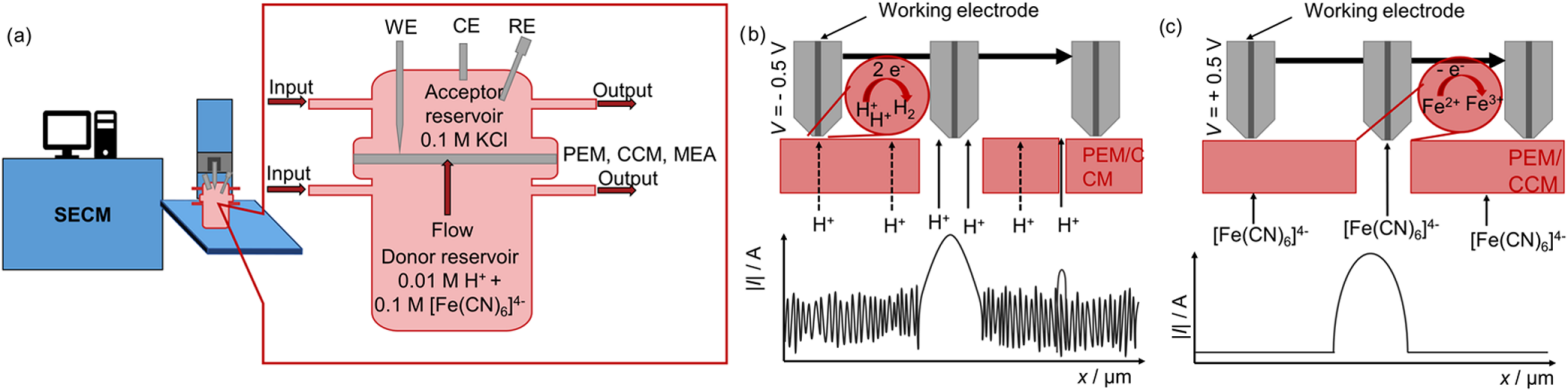
(a) Principle assembly of the DiffC-DC-SECM setup with the enlarged flow-through diffusion cell. Measurement principle for (b) the determination of the through-plane proton conductivity with protons as a redox mediator and (c) the detection of perforating defects with ferrocyanide as a redox mediator.

Since ferrocyanide cannot diffuse through an intact membrane, a pristine membrane shows at anodic bias a low current response due to both the insulating properties of the membrane and the prevention of mediator diffusion, which is referred to as negative feedback (Figure S3a).^32–34^

In contrast, if perforating membrane defects are present, ferrocyanide diffuses through these defects, resulting in a current increase when the tip scans over such a structural defect (Figures 2c and S3b), which is characterized as positive feedback.

This approach thus enables clear discrimination between intact and damaged membrane areas and differentiation from functional impairments such as local proton conductivity changes. To compare and validate the IC-AC-SECM and DiffC-DC-SECM methods for the characterization of different aging phenomena, measurements are conducted on PEM-AH, CCM-AH, CCM-AA, and CCM-RA samples.

### 3.1 IC-AC-SECM Measurements

The samples PEM-AH and CCM-AH were first investigated by SEM in order to check the successful preparation of the two artificial holes. As a result, two distinct holes with a length of approximately 110 μm and a distance of 1.2 mm are clearly observable for the PEM-AH (Figure 3a). As for the CCM-AH, the two holes are characterized by lengths between around 100 and 230 μm with a spacing of 1.2 mm (Figure 3b).

**Figure 3:**
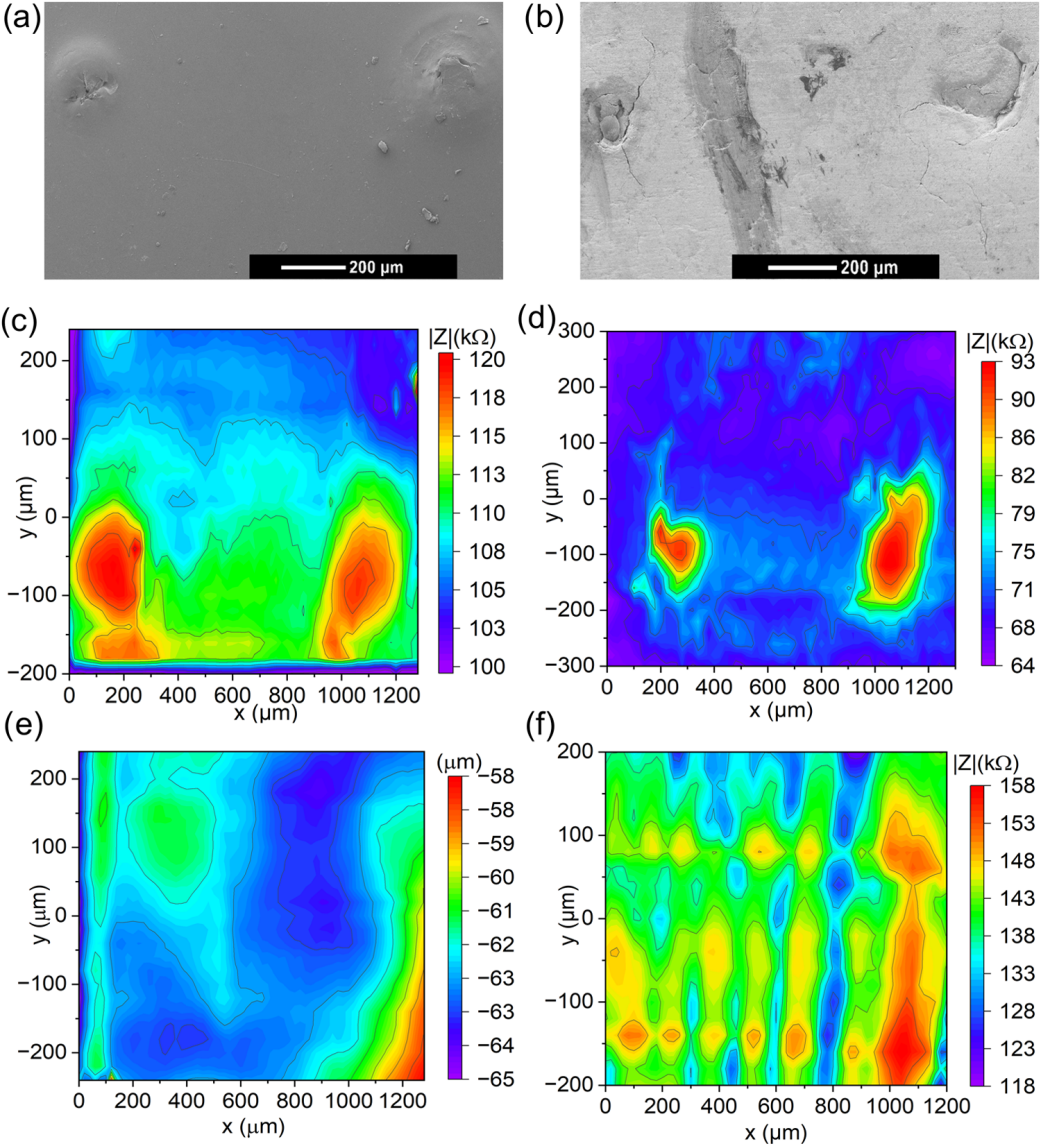
SEM images of the: (a) PEM-AH and (b) CCM-AH. IC-AC-SECM area scans: (c) Impedance of the PEM-AH, (d) impedance of the CCM-AH, (e) topography of the PEM-AH, and (f) impedance of the CCM-AA.

In the IC-AC-SECM image shown in Figure 3c, the areas around [*x* = 150 μm, *y* = −70 μm] and [*x* = 1100 μm, *y* = −90 μm] exhibit a higher impedance. At the locations of the holes, the impedance is expected to be higher compared with the intact membrane, as the underlying poly­(tetrafluoroethylene) sample holder, characterized by a higher impedance, influences the measured signal. The regions of increased impedance have lengths of about 100 to 150 μm and are spaced approximately 1 mm apart, which corresponds well to the dimensions of the holes as observed in the SEM image. As mentioned above, IC-AC-SECM should be able to measure the topography of the sample separately, and pinholes would be expected to lead to topographic unevenness in the membrane. Figure 3e presents the topography of the PEM-AH sample after tilt correction. The data indicate the absence of distinct height differences in the regions of the expected holes. This may be due to the soft and thin nature of the membrane, which likely impairs accurate topographical resolution.

The IC-AC-SECM image of the CCM-AH sample in Figure 3d shows a smaller area around [*x* = 250 μm, *y* = −100 μm] and a larger area around [*x* = 1050 μm, *y* = −100 μm] with increased impedance. These regions exhibit lengths of about 100 and 200 μm, respectively, and are spaced approximately 1 mm apart, which also corresponds well to the holes observed in the SEM image. Interestingly, the overall impedance across the CCM surface is approximately half that measured over the PEM. This observation is primarily attributed to the Pt/C catalyst layer coating on the CCM, which enhances its electronic conductivity compared to the PEM and thus obviously leads to a lower overall impedance.

To further investigate the method’s capability to detect more realistic aging mechanisms, the CCM-AA sample was examined. The formation of perforative defects in single PEMs induced by treatment with the Fenton reagent has previously been demonstrated.^27,35,36^ As for the Fenton reagent-treated CCM, several regions of very high impedance at [*x* = 1000 μm, −200 μm < *y* < 150 μm] and moderately increased impedance at [0 μm < *x* < 700 μm, *y* = −150 and 100 μm] are evident (Figure 3f). In general, the impedance distribution across the surface appears to be highly inhomogeneous. Notably, the impedance values are generally higher than those observed for the pristine CCM. This suggests that the CCM’s conductivity decreased after Fenton degradation. However, due to limitations inherent to the IC-AC-SECM measurement technique, it is not possible to distinguish whether areas of increased impedance arise from structural defects in the membrane, an eroded catalyst or carbon support layer, or from a reduced proton conductivity of the membrane. The topographical data obtained from the IC-AC-SECM measurements of the investigated CCMs also did not reveal any clear evidence for structural defects (Figure S1).

### 3.2 DiffC-DC-SECM Measurements

In the next step, the PEM-AH sample was characterized using the novel DiffC-DC-SECM technique. Figure 4a shows that at negative bias (with protons as addressed redox mediator), the negative current within the range of [*x* = 400 μm, −250 μm < *y* < −50 μm] and [*x* = 1650 μm, *y* = −100 μm (and above)] has increased more than 10-fold compared to the area between these two conspicuous regions. The corresponding area scan at positive bias with ferrocyanide as the redox mediator reveals the highest currents at about [*x* = 400 μm, −250 < *y* < 100 μm] and [*x* = 1650 μm, *y* = −150 μm and *y* > −50 μm] (Figure 4b), which corresponds with the positions of high (negative) currents in the proton scan. Ferrocyanide can diffuse only through perforative defects, where it is oxidized at the WE. The use of two redox-active species, simultaneously present in the lower part of the cell, along with the avoidance of disassembly, allows the coordinates of the holes to be reproduced almost exactly. Furthermore, the impedance peculiarities identified as two holes are well-separated and spatially resolved as diffusion is minimized by using the flow-through cell approach. It is known that ferrocyanide can decompose upon the addition of strong acids. However, the pH of the donor solution was moderately acidic at 3.54. In addition, long-term stability tests were conducted. Cyclic voltammograms recorded over a period of 5 h demonstrated excellent stability of the solution, allowing the potential decomposition of the complex to be ruled out (Figure S4).

**Figure 4:**
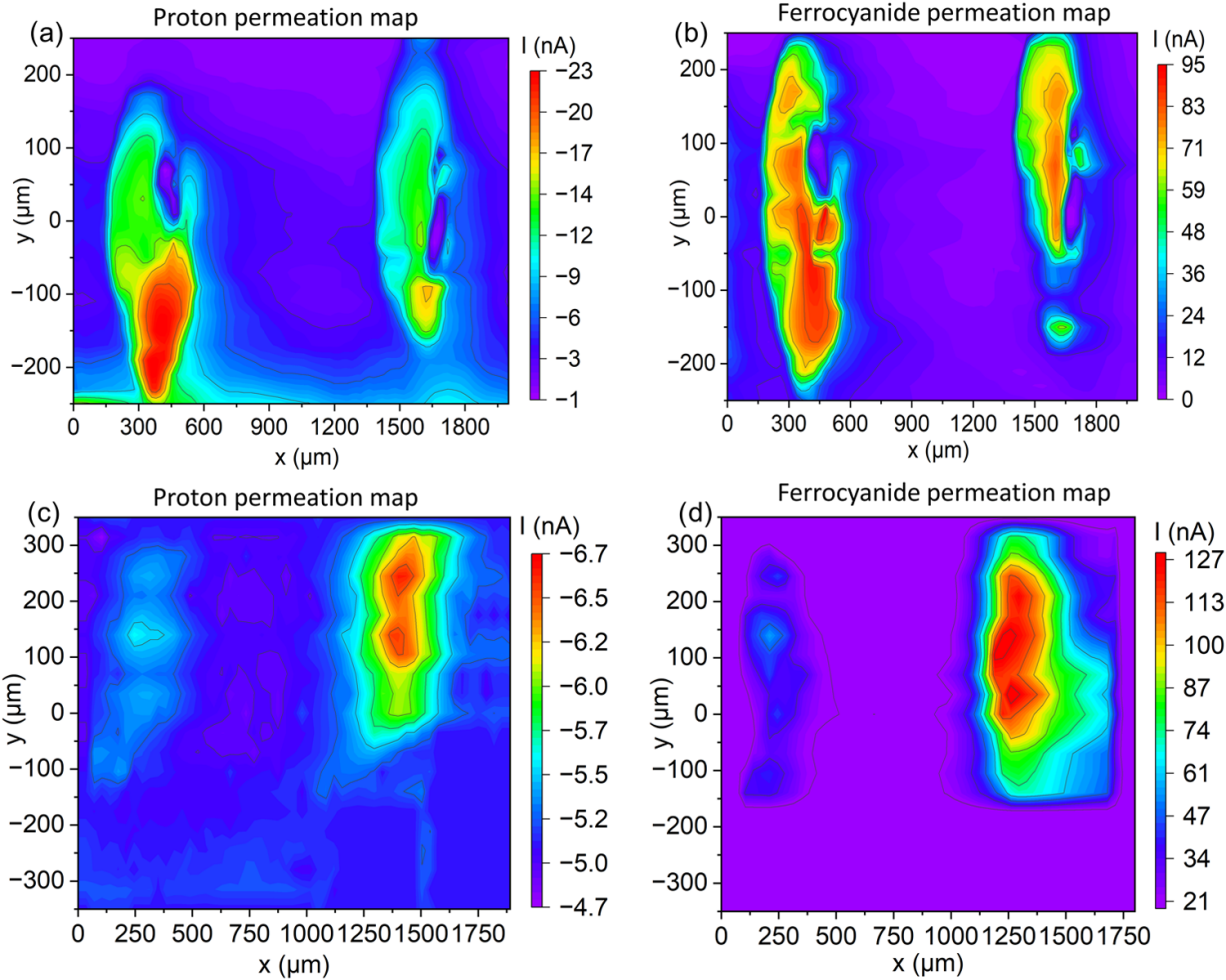
DiffC-DC-SECM area scans of the: (a) PEM-AH at negative bias (protons as the redox mediator), (b) PEM-AH at positive bias (ferrocyanide as the redox mediator), (c) CCM-AH at negative bias (protons as the redox mediator), and (d) CCM-AH at positive bias (ferrocyanide as the redox mediator).

The size of the holes observed in the DiffC-DC-SECM measurements in the *x*-direction, as well as the distance between the holes, is very well comparable with those identified by the IC-AC-SECM and SEM measurements. In the *y*-direction, the holes appear to be elongated. In part, this is due to the different scaling of the *x*- and *y*-axes. The difference in the IC-AC-SECM images can be attributed to diffusion effects in the DiffC-DC-SECM technique, which can blur the boundaries of the features and lead to an overestimation of their lateral dimensions.

In addition, the CCM-AH sample was characterized with the DiffC-DC-SECM approach, and as a result, the proton current reaches its absolute maximum in the range of [*x* = 250 μm, *y* = 50 μm] and [*x* = 1400 μm, 100 μm < *y* < 300 μm] (Figure 4c). The highest absolute currents using ferrocyanide as the redox mediator were measured at nearly the same coordinates (Figure 4d). Since the diffusion of ferrocyanide is only possible through perforative defects, it can be concluded with certainty that the increased iron current in these regions indicates structural transmembrane defects. The results are also in good agreement with the corresponding IC-AC-SECM and SEM images, where a small and a larger hole could be detected.

Furthermore, chemically aged CCM samples have been investigated to simulate more realistic deactivation. The SEM analysis of the Fenton reagent-aged CCM-AA sample (Figure 5b,c) reveals a markedly more heterogeneous surface compared to a pristine CCM (Figure 5a). Surface imaging indicates cracks within the catalyst layer. However, based on these images alone, it is not possible to determine whether such defects also extend into the PEM or are confined to the catalyst layer.

**Figure 5:**
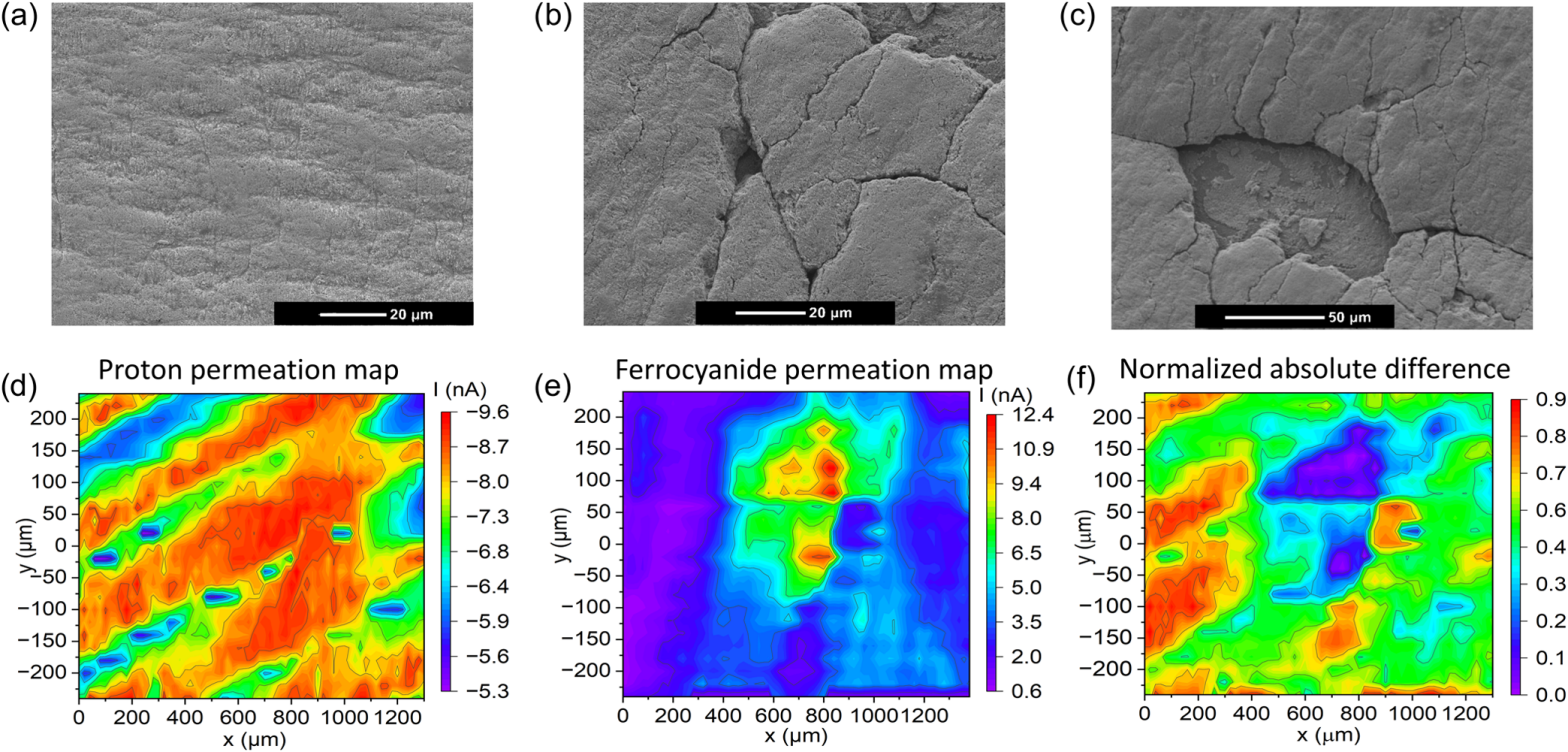
SEM images of the (a) pristine CCM, and (b, c) CCM-AA. DiffC-DC-SECM area scans of the CCM-AA: (d) At negative bias (protons as the redox mediator), (e) at positive bias (ferrocyanide as the redox mediator), (f) an absolute normalized difference image of both DiffC-DC-SECM area scans shown in panels (d, e). The purple-blue region clearly indicates perforative defects, while the yellow-red areas are due to an increased through-plane proton conductivity that cannot be explained by transmembrane perforations, likely due to a thinned PEM caused by chemical degradation.

The DiffC-DC-SECM scan of the CCM-AA in Figure 5d shows extended regions of elevated negative current at negative bias across the surface caused by the reduction of protons. A wave-like pattern of high and low current density can be observed. The corresponding DiffC-DC-SECM area scan at positive bias in Figure 5e shows a more separated region of high positive current, particularly in the area at [500 μm < *x* < 900 μm, −50 μm < *y* < 200 μm], which is attributed to an elevated iron concentration. At least six distinct well-separated current hot spots centered at *y* = −20 μm, *y* = 70 μm, *y* = 120 μm, and *y* = 180 μm are clearly discernible here. Obviously, perforative membrane defects have formed at these positions due to the Fenton reaction. The dimensions of the perforative defects exhibit lengths and widths spanning from 20 to 150 μm. The achieved detection of separated holes with sizes in the range of the UME tip diameter indicates that the resolution is not limited here by diffusion effects.

To enable a more accurate comparison between the two scans, both data sets were normalized to the highest current value in the respective measurement. This position is assumed to indicate the presence of perforations in the membrane. Subsequently, the absolute difference between the two normalized scans is calculated. Regions with a value of zero indicate agreement between the scans, suggesting the presence of perforative defects. Deviations from zero reflect variations in the through-plane proton conductivity alone. In the difference image shown in Figure 5f, two discernible regions centered at [*x* = 750 μm, *y* = −30 μm] and [*x* = 700 μm, *y* = 120 μm] exhibit minimal or zero difference between the two scans. The observed correspondence between the measurements suggests the presence of structural perforative defects, such as cracks and holes, in this region. The areas in the difference image with values above zero represent inhomogeneities in proton conductivity that cannot be attributed to cracks or holes. This may suggest localized membrane thinning due to a partial chemical degradation of the PEM by the Fenton reagent, which results in an increased through-plane proton permeability at these positions.^37^ Consequently, the differential scan image clearly demonstrates the ability of the DiffC-DC-SECM technique to distinguish between perforative defects and changes in functional membrane properties due to the local heterogeneous proton conductivity.

Finally, the CCM-RA sample was investigated after undergoing standardized AST to test the chemical stability of membranes in the fully assembled PEMFCs. In the procedure, the fuel cell is maintained at an OCV for 24 h, followed by a regeneration step and subsequent electrochemical analysis. This AST and analysis cycle is repeated over the course of 500 h. Maintaining the cell at OCV promotes the formation of reactive species, which are known to degrade the membrane structure through radical-induced attack.^38^ Selected electrochemical measurement data of the AST are presented in the Supporting Information (Figures S5 and S6). LSV measurements reveal an increase in hydrogen crossover current from 6.28 mA cm^–2^ at the beginning of the test to 12.33 mA cm^–2^ over the course of the aging process, indicating a progressive loss of membrane integrity. After completion of the test, a thermography measurement was performed, which revealed a localized temperature hotspot at the position of the flow-field hydrogen gas inlet (Figure 6a). Both findings suggest the presence of structural perforative defects in the membrane, which allow hydrogen (or protons) to diffuse through these defects. SEM imaging of the CCM-RA sample after the AST indicates pronounced structural changes in the region of the hydrogen inlet, where the catalyst layer appears to be roughened and damaged (Figure 6b). In contrast, the central area of the CCM-RA, where thermography measurements did not indicate increased hydrogen permeability, exhibits only minor surface alterations and appears overall to be less degraded (Figure 6c).

**Figure 6:**
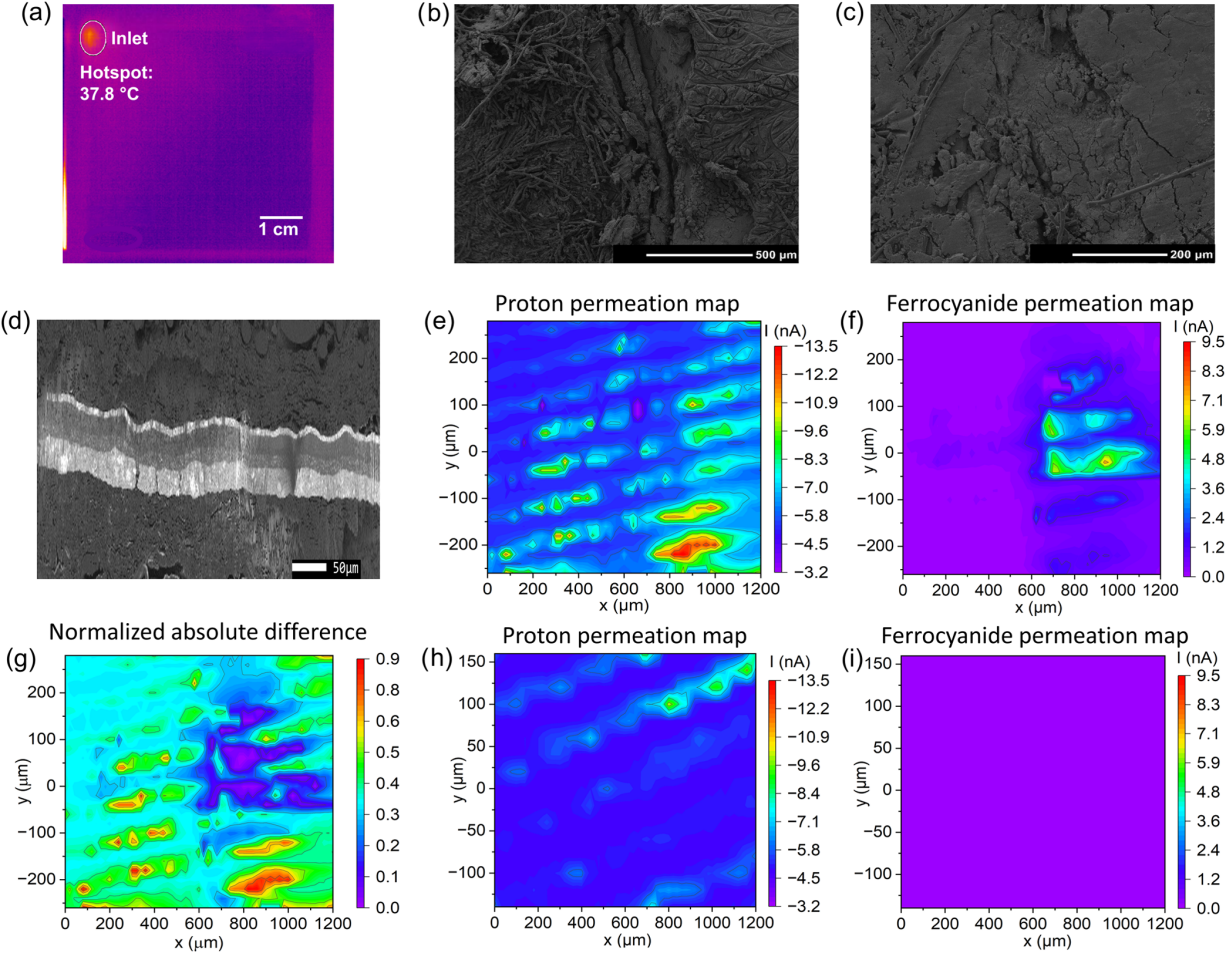
(a) Thermographic image of the OCV AST aged MEA. SEM images of the: (b) CCM-RA at the former position of the hydrogen gas inlet region, (c) CCM-RA outside the hydrogen gas inlet region, and (d) cross-section of a pristine MEA. DiffC-DC-SECM area scans of the CCM-RA in the former region of the hydrogen gas inlet: (e) At negative bias (protons as the redox mediator), (f) at positive bias (ferrocyanide as the redox mediator), and (g) absolute difference image of both DiffC-DC-SECM area scans. DiffC-DC-SECM area scans in the center of the CCM-RA outside the hydrogen gas inlet region: (h) At negative bias (protons as the redox mediator) and (i) at positive bias (ferrocyanide as the redox mediator). Note that in (h, i), the same current scaling as in (e, f) was applied for optimized comparison.

DiffC-DC-SECM measurements were performed in the area of the suspect region around the previous hydrogen gas inlet position in order to clarify the exact nature and shape of possible membrane defects. Figure 6e reveals a wave-like pattern of elevated negative current at negative bias (i.e., due to proton reduction). Overall, the proton conductivity across the surface exhibits higher spatial variation in the CCM-RA compared to the pristine sample. Figure 6f displays four larger and several smaller isolated regions of elevated positive current at positive bias (i.e., due to iron­(II) oxidation) within the range of [700 μm < *x* < 1100 μm, −50 μm < *y* < 150 μm]. The defects exhibit lengths and widths from 20 μm to 260 μm. This provides evidence for the presence of structural perforative defects. For better comparability of the scans, the absolute difference image was calculated. Figure 6g shows that in the region at [700 μm < *x* < 1100 μm, −50 μm < *y* < 150 μm], the difference between the two scans approaches zero, proving the presence of several holes and cracks in this region. Furthermore, in the regions [100 μm < *x* < 500 μm, −250 μm < *y* < 50 μm] and [700 μm < *x* < 1000 μm, −240 μm < *y* < −120 μm], partially high differences are observable, indicating increased proton conductivity. These differences, however, occur in the obvious absence of transmembrane perforative defects, which may indicate a locally thinned membrane that could lead to perforative defects as aging progresses.

For comparison, an additional DiffC-DC-SECM measurement was performed at the center of the same CCM-RA sample (Figure 6h), where the thermography did not reveal an increased hydrogen permeability. It can be observed that the proton conductivity across the surface is, on average, lower than in the region around the inlet. Higher absolute currents are again observed in a wave-like pattern with the highest absolute values at the region around [700 μm < *x* < 1200 μm, 75 μm < *y* < 150 μm]. The corresponding area scan detecting ferrocyanide as a redox mediator does not show a comparable pattern or regions with altered absolute current (Figure 6i). This finding suggests that the increased negative current observed under negative bias is not due to existing structural perforative defects, but may be linked to a locally thinned membrane.

It has been repeatedly reported that membrane degradation in PEMFCs is particularly pronounced in the region of the hydrogen gas inlet.^39–42^ This accelerated degradation of the PEM can be attributed to the higher partial pressure of hydrogen and the resulting increase in hydrogen crossover. The intensified formation of radicals in this area leads to membrane thinning, which in turn causes a further increase in hydrogen permeability.^39–42^ SECM measurements revealed strong membrane damage in the region of the hydrogen inlet. Several holes and a thin membrane were identified. Complementary to thermographic analysis and hydrogen crossover current measurements, DiffC-DC-SECM enables detailed characterization of the location, number, size, and structure of the defects and can even visualize precursor states, such as a thinned membrane before a perforative crack or hole has formed. These findings allow for deeper insights into the aging phenomena of the membrane, facilitating a more comprehensive understanding of localized degradation processes.

Interestingly, a peculiar diagonal wave-like pattern with typical distances between the “wave crests” of about 50 μm was observed in nearly all SECM scans of CCM samples (cf. Figures 3f, 5d, 6e, and 6h). A similar feature was also identified in the cross-sectional SEM image of the investigated MEA with a wave-like structure on the anode side at the catalyst layer/PEM interface with a typical distance between the “wave crests” of 30 to 45 μm (Figure 6d). It has been reported that during the fabrication of the CCM, the PEM may swell or deform due to the catalyst coating process, potentially leading to surface irregularities.^43^ Obviously, these structural imperfections can subsequently be detected by SECM, i.e., the increased proton conductivity at the “wave crests” in the DiffC-DC-SECM scans is a result of a topographic wave valley with a decreased PEM thickness.

Independent IC-AC-SECM measurements of both PEM and CCM samples were performed prior to the DiffC-DC-SECM experiments, in which the tilt-corrected topography of the sample surface was recorded (cf. Figures 3e and S1). In these measurements, no indication of the described wave-like pattern was observed. This suggests that the observed conductivity variations in the DiffC-DC-SECM scans are not due to surface undulations affecting the tip–sample distance. In addition, all samples were continuously stored in ultrapure water and remained fully hydrated at all times. Membrane swelling caused by exposure to the measurement electrolyte can thus be excluded. These findings support the conclusion that the wave-like features originate from the internal structure of the CCM, likely introduced during its fabrication process, and not from postprocessing or measurement-related effects.

## 4 Conclusions

In this study, two spatially resolved electrochemical methods, IC-AC-SECM and the novel diffusion-cell coupled DC-SECM technique, were evaluated for their suitability in characterizing different degradation phenomena in PEMs, CCMs, and MEAs. To achieve this, a range of samples was investigated, including pristine PEMs with artificial holes, CCMs with artificial holes, CCMs subjected to artificial chemical aging, and MEAs in fully assembled fuel cells aged using the DoE-proposed OCV AST for the standardized investigation of chemical membrane aging in PEMFCs. It was found that the IC-AC-SECM method successfully identified artificially introduced holes in both the PEM and CCM samples. However, it was unable to effectively characterize the degradation in the chemically aged CCM, as it could not differentiate between local topographic and functional differences. With the DiffC-DC-SECM configuration, it was demonstrated that the technique can not only detect perforative defects in isolated PEMs, but that it can also discriminate functional and structural defects in membranes of chemically aged 3-layer MEAs (i.e., CCMs) with high spatial resolution. Moreover, the method is also able to identify perforative membrane defects as well as thinned membrane regions with altered through-plane conductivity of CCMs aged in fully assembled and operational fuel cells. These findings are in perfect agreement with the results of electrochemical measurements revealing an increased hydrogen crossover current and thermographic imaging showing a pronounced gas breakthrough of the MEA in the region of the hydrogen gas inlet. Compared with these conventional methods, DiffC-DC-SECM offers higher spatial resolution and greater sensitivity to local electrochemical changes. It therefore proves to be a valuable tool for assessing membrane integrity and can help to enlighten membrane damage already at an early stage before it becomes critical for operation. A key advantage of this method lies in its capacity to detect localized aging phenomena in PEMs on the micrometer scale, even in catalyst-coated membranes. This makes it especially suitable for the in-depth pre- and post-mortem analysis of membranes in complete MEAs and fully assembled fuel cells (or electrolyzers) before and after operation and as a tool for quality control in MEA manufacturing processes. Hence, the method can open up new vistas for predictive diagnostics and targeted durability assessments in fuel cell and electrolyzer research and development.

While the present study focuses on micrometer-scale defects, such as membrane perforations and localized thinning, the detection of nanoscale mechanical damage remains an important challenge for future work. These smaller defects are highly relevant to real-world degradation but require different experimental conditions and instrumentation. In this study, the electrode dimensions were deliberately chosen to match the expected size range of artificial and operationally induced defects, ensuring robust signal and spatial resolution for the intended application. Addressing nanoscale damage would require the implementation of significantly smaller ultramicroelectrodes and optimized noise reduction strategies. The potential value of such high-resolution measurements is recognized, and the development of nanoscale-capable SECM setups represents a promising direction for future investigations aimed at early stage membrane failure diagnostics. Moreover, the integration of numerical modeling tools such as COMSOL Multiphysics represents a valuable opportunity for future studies.^44^ In particular, such simulations may be beneficial for validating spatial profiles, optimizing electrode design, and reconstructing local concentration fields. Therefore, the development of those models represents a logical and promising next step to further enhance the analytical accuracy and applicability of the DC-SECM technique in membrane diagnostics.

## Supplementary Material



## Notes

IC-AC-SECM area scans: topography of the CCM-AH and topography of the CCM-AA (Figure S1); detailed experimental description of the RG ratio calculation; cyclic voltammograms as used to determine the radius of the platinum ultramicroelectrode, microscopy image of the platinum ultramicroelectrode to evaluate the RG ratio (line length = 382 μm) (Figure S2); detailed experimental description of the approach curve measurement; approach curves above a catalyst-coated membrane at an intact site (negative feedback at positive bias and positive feedback at negative bias) and a perforated site (positive feedback at both biases) (Figure S3); detailed experimental description of the CV measurement for the donor solution stability; cyclic voltammograms of the donor solution recorded over a period of 5 h (Figure S4); detailed experimental description of the OCV AST with results of the electrochemical characterization; illustration of the voltage over the complete duration of the OCV AST including the recovery protocol (Figure S5); polarization curve at the beginning and end of the OCV AST (Figure S6); and gas flow configuration for the recovery protocol (Table S1) (PDF)

## References

[1] European Commission . Directorate General for Mobility and Transport. In EU Transport in Figures: Statistical Pocketbook 2024; Publications Office: LU, 2024.

[2] Haasz T., Gómez Vilchez J. J., Kunze R., Deane P., Fraboulet D., Fahl U., Mulholland E. (2018). Perspectives
on decarbonizing the transport sector in the EU-28. Energy Strategy Rev..

[3] Ogungbemi E., Wilberforce T., Ijaodola O., Thompson J., Olabi A. (2021). Selection
of proton exchange membrane fuel cell for transportation. Int. J. Hydrogen Energy.

[4] Marcinkoski, J. ; Vijayagopal, R. ; Adams, J. ; James, B. ; Kopasz, J. ; Ahluwalia, R. Hydrogen Class 8 Long Haul Truck Targets Department of Energy 2019.

[5] Staffell I., Scamman D., Abad A. V., Balcombe P., Dodds P. E., Ekins P., Shah N., Ward K. R. (2019). The role of hydrogen
and fuel cells in the global energy system. Energy Environ. Sci..

[6] Fan L., Tu Z., Chan S. H. (2021). Recent development of hydrogen and fuel cell technologies:
A review. Energy Rep..

[7] Babic U., Suermann M., Büchi F. N., Gubler L., Schmidt T. J. (2017). Critical
Review—Identifying Critical Gaps for Polymer Electrolyte Water
Electrolysis Development. J. Electrochem. Soc..

[8] Honsho Y., Nagayama M., Matsuda J., Ito K., Sasaki K., Hayashi A. (2023). Durability of PEM water electrolyzer
against wind power
voltage fluctuation. J. Power Sources.

[9] Zatoń M., Rozière J., Jones D. J. (2017). Current understanding of chemical
degradation mechanisms of perfluorosulfonic acid membranes and their
mitigation strategies: a review. Sustainable
Energy Fuels.

[10] Zhang H., Shen P. K. (2012). Recent Development
of Polymer Electrolyte Membranes
for Fuel Cells. Chem. Rev..

[11] Cullen D. A., Neyerlin K. C., Ahluwalia R. K., Mukundan R., More K. L., Borup R. L., Weber A. Z., Myers D. J., Kusoglu A. (2021). New roads
and challenges for fuel cells in heavy-duty transportation. Nat. Energy.

[12] Ren P., Pei P., Li Y., Wu Z., Chen D., Huang S. (2020). Degradation
mechanisms of proton exchange membrane fuel cell under typical automotive
operating conditions. Prog. Energy Combust.
Sci..

[13] Kelly M., Egger B., Fafilek G., Besenhard J., Kronberger H., Nauer G. (2005). Conductivity of polymer
electrolyte
membranes by impedance spectroscopy with microelectrodes. Solid State Ionics.

[14] Wang X., Qi J., Ozdemir O., Uddin A., Pasaogullari U., Bonville L. J., Molter T. (2014). Ca^2+^ as
an Air Impurity
in Polymer Electrolyte Membrane Fuel Cells. J. Electrochem. Soc..

[15] Wu J., Yuan X. Z., Martin J. J., Wang H., Zhang J., Shen J., Wu S., Merida W. (2008). A review of PEM fuel
cell durability: Degradation mechanisms and mitigation strategies. J. Power Sources.

[16] Cheng X., Shi Z., Glass N., Zhang L., Zhang J., Song D., Liu Z.-S., Wang H., Shen J. (2007). A review of PEM hydrogen
fuel cell contamination: Impacts, mechanisms, and mitigation. J. Power Sources.

[17] Sugawara T., Kawashima N., Murakami T. N. (2011). Kinetic study of Nafion degradation
by Fenton reaction. J. Power Sources.

[18] Okonkwo P. C., Belgacem I. B., Emori W., Uzoma P. C. (2021). Nafion degradation
mechanisms in proton exchange membrane fuel cell (PEMFC) system: A
review. Int. J. Hydrogen Energy.

[19] Tang H., Peikang S., Jiang S. P., Wang F., Pan M. (2007). A degradation
study of Nafion proton exchange membrane of PEM fuel cells. J. Power Sources.

[20] Hongsirikarn K., Mo X., Goodwin J. G., Creager S. (2011). Effect of H2O2 on Nafion properties
and conductivity at fuel cell conditions. J.
Power Sources.

[21] Lin R., Li B., Hou Y., Ma J. (2009). Investigation of dynamic driving
cycle effect on performance degradation and micro-structure change
of PEM fuel cell. Int. J. Hydrogen Energy.

[22] Lim C., Ghassemzadeh L., Van Hove F., Lauritzen M., Kolodziej J., Wang G., Holdcroft S., Kjeang E. (2014). Membrane degradation
during combined chemical and mechanical
accelerated stress testing of polymer electrolyte fuel cells. J. Power Sources.

[23] Chandesris M., Vincent R., Guetaz L., Roch J.-S., Thoby D., Quinaud M. (2017). Membrane degradation
in PEM fuel cells: From experimental
results to semi-empirical degradation laws. Int. J. Hydrogen Energy.

[24] Chen Y., Bahrami M., Kumar N., Orfino F. P., Dutta M., Lauritzen M., Setzler E., Agapov A. L., Kjeang E. (2023). Effect of
Accelerated Stress Testing Conditions on Combined Chemical and Mechanical
Membrane Durability in Fuel Cells. J. Electrochem.
Soc..

[25] Ramani D., Singh Y., Orfino F. P., Dutta M., Kjeang E. (2018). Characterization
of Membrane Degradation Growth in Fuel Cells Using X-ray Computed
Tomography. J. Electrochem. Soc..

[26] Thiel, S. ; Seiß, V. ; Eichelbaum, M. In Scanning Electrochemical Microscopy for the Characterization of Fuel Cell Components, International Workshop on Impedance Spectroscopy (IWIS); IEEE, 2022; pp 14–19.

[27] Thiel S., Eichelbaum M. (2024). Scanning electrochemical
microscopy for the differentiation
of radical-induced degradation mechanisms in polymer electrolyte membranes. RSC Adv..

[28] Abdel-Baset, T. ; Benjamin, T. ; Borup, R. Fuel Cell Technical Team Roadmap Driving Research and Innovation for Vehicle Efficiency and Energy Sustainability Partnership 2017.

[29] Catarelli S. R., Lonsdale D., Cheng L., Syzdek J., Doeff M. (2016). Intermittent
Contact Alternating Current Scanning Electrochemical Microscopy: A
Method for Mapping Conductivities in Solid Li Ion Conducting Electrolyte
Samples. Front. Energy Res..

[30] Bio-Logic Science Instruments *Intermittent Contact (ic) SECM for relief of major topographic features*; 2016.

[31] Eckhard K., Schuhmann W. (2008). Alternating
current techniques in scanning electrochemical
microscopy (AC-SECM). Analyst.

[32] Bertoncello P. (2010). Advances on
scanning electrochemical microscopy (SECM) for energy. Energy Environ. Sci..

[33] Polcari D., Dauphin-Ducharme P., Mauzeroll J. (2016). Scanning Electrochemical Microscopy:
A Comprehensive Review of Experimental Parameters from 1989 to 2015. Chem. Rev..

[34] Mader J. A., Benicewicz B. C. (2011). Synthesis and Properties of Segmented
Block Copolymers
of Functionalised Polybenzimidazoles for High-Temperature PEM Fuel
Cells. Fuel Cells.

[35] Shi W., Baker L. A. (2015). Imaging heterogeneity
and transport of degraded Nafion
membranes. RSC Adv..

[36] Teixeira F. C., Teixeira A. P., Rangel C. (2023). Chemical stability
of new nafion
membranes doped with bisphosphonic acids under Fenton oxidative conditions. Int. J. Hydrogen Energy.

[37] Kwon J., Jo S., Cho K.-Y., Eom K. (2020). Deconvolution of the dehydration
degradation mechanism in polymer electrolyte membrane fuel cells using
electrochemical impedance analysis combined with the transmission
line model under low humidity. J. Power Sources.

[38] Rodgers M. P., Bonville L. J., Kunz H. R., Slattery D. K., Fenton J. M. (2012). Fuel Cell
Perfluorinated Sulfonic Acid Membrane Degradation Correlating Accelerated
Stress Testing and Lifetime. Chem. Rev..

[39] Yu J., Matsuura T., Yoshikawa Y., Nazrul Islam M., Hori M. (2005). Lifetime behavior of a PEM fuel cell
with low humidification of feed
stream. Phys. Chem. Chem. Phys..

[40] Koprek M., Schlumberger R., Wachtel C., Wilhelm F., Messerschmidt M., Scholta J., Hölzle M. (2022). Local ageing effects of polymer electrolyte
fuel cell membrane electrode assemblies due to accelerated durability
testing. Fuel Cells.

[41] De
Moor G., Bas C., Charvin N., Dillet J., Maranzana G., Lottin O., Caqué N., Rossinot E., Flandin L. (2016). Perfluorosulfonic
acid membrane degradation in the hydrogen inlet region: A macroscopic
approach. Int. J. Hydrogen Energy.

[42] Schoemaker M., Misz U., Beckhaus P., Heinzel A. (2014). Evaluation of Hydrogen
Crossover through Fuel Cell Membranes. Fuel
Cells.

[43] Mo S., Du L., Huang Z., Chen J., Zhou Y., Wu P., Meng L., Wang N., Xing L., Zhao M., Yang Y., Tang J., Zou Y., Ye S. (2023). Recent Advances
on PEM Fuel Cells: From Key Materials to Membrane Electrode Assembly. Electrochem. Energy Rev..

[44] Ye Z., Zhu Z., Zhang Q., Liu X., Zhang J., Cao F. (2018). In situ SECM
mapping of pitting corrosion in stainless steel using submicron Pt
ultramicroelectrode and quantitative spatial resolution analysis. Corros. Sci..

