# Supplementary material for: Spatially Resolved Differentiation of Functional Degradation and Perforating Structural Defects in Membrane Electrode Assemblies Using Diffusion-Cell Coupled DC-SECM
Source: ACS Meas Sci Au. 2025 Aug 12;5(5):740–50. doi: 10.1021/acsmeasuresciau.5c00071 (PMC12532053; doi:10.1021/acsmeasuresciau.5c00071)
Supplement: Supplementary file 1 [file tg5c00071_si_001.pdf]

Supporting Information

Spatially Resolved Differentiation of Functional Degradation and Perforating Structural Defects in Membrane Electrode Assemblies Using Diffusion-Cell Coupled DC-SECM

Susanne Thiel and Maik Eichelbaum*

Institute for Applied Hydrogen Research, Electro- and Thermochemical Energy Systems (H2Ohm), Technische Hochschule Nürnberg Georg Simon Ohm, Prinzregentenufer 47, 90489 Nuremberg, Germany

IC-AC-SECM

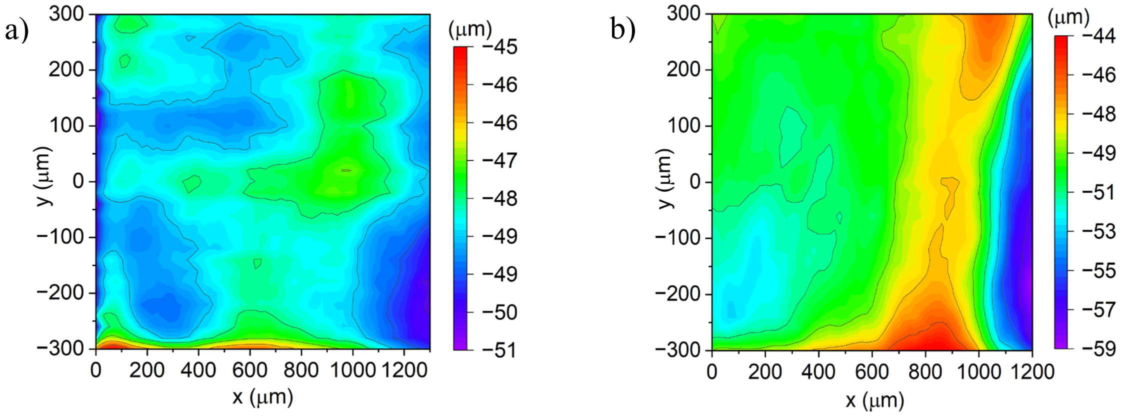

Figure S1: Tilt-corrected topographical IC-AC-SECM area scans: a) CCM-AH, b) CCM-AA.

DiffC-DC-SECM

Prior to determining the RG ratio, the platinum ultramicroelectrode was electrochemically cleaned by performing 10 cyclic voltammetry cycles in 100 mM sulfuric acid, scanning between 0 and 1.2 V at a rate of 10 mV s^{-1}. Subsequently, a cyclic voltammogram was recorded in a 0.1 M aqueous solution containing potassium ferrocyanide, potassium ferricyanide, and potassium chloride (all analytical grade, Carl Roth) to determine the RG ratio.
The measurement was performed using the SECM setup, applying a potential range from -0.30 V to +0.65 V with a sweep rate of 0.025 V s^{-1}. A platinum wire ultramicroelectrode (UME) with a nominal diameter of 15 µm was used as the working electrode (WE), an Ag/AgCl reference electrode (3 M KCl, RE-1CP), and a platinum sheet as the counter electrode (CE) (all from BioLogic). Using the steady-state current and the equation for UMEs, the radius of the platinum tip was determined, which allows for calculation of the RG ratio.[1]

$$d = \frac{2i_{ss}}{4nFDC}$$

C= 96485 C mol^{-1}; D= 6.81*10^{-6} cm^2 s^{-1}; c = 1*10^{-4} mol cm^{-1}; I_{ss}= 202.3*10^{-9} A

$$d = 15.4\ \mu m$$

It was shown that the diameter of the platinum wire, determined via cyclic voltammetry, was 15.4 µm, confirming the manufacturer's specification. The diameter of the surrounding glass capillary was measured using the camera microscopy system of a micro-XRF instrument (XGT-9000, Horiba) and determined to be 382 µm. This results in an RG ratio of 24.8.

$$RG = \frac{R}{r}$$

$$RG = 24.8$$

a) b)

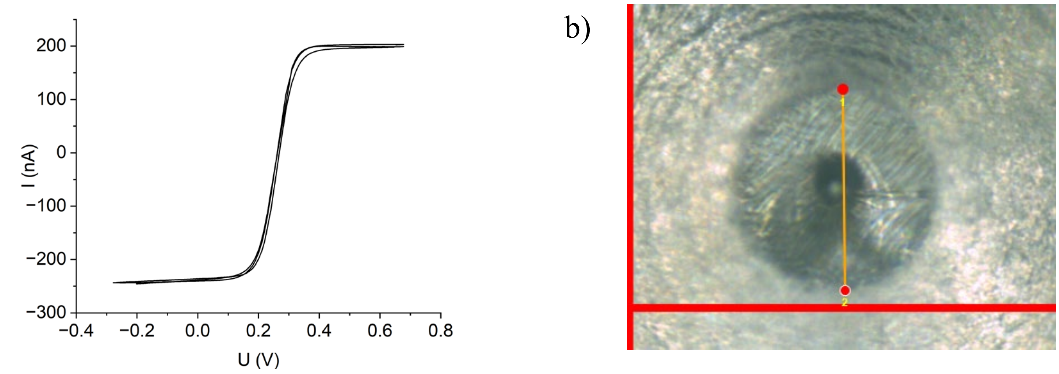

Figure S2: a) Cyclic voltammograms as used to determine the radius of the platinum ultramicroelectrode, b) microscopy image of the platinum ultramicroelectrode to evaluate the RG ratio (line length = 382 µm).

Furthermore, approach curves were recorded above a catalyst-coated membrane (CCM) at both an intact region and a perforated defect. The measurements were conducted at potentials of +0.5 V and –0.5 V with a step size of 50 µm and a scan rate of 50 µm s^{-1}. As shown in Figure S3a, negative feedback is observed at a positive bias when approaching the intact CCM. This behavior is attributed to the fact that ferrocyanide ions cannot permeate the intact membrane, and only potassium chloride is present in the acceptor solution. In contrast, at a negative bias, positive feedback is detected, as protons can diffuse through the intact membrane and are reduced at the working electrode. At the perforated region (Figure S3b), positive feedback is observed at both +0.5 V and –0.5 V. This is due to the increasing local concentration of the iron species and protons at the defect site.

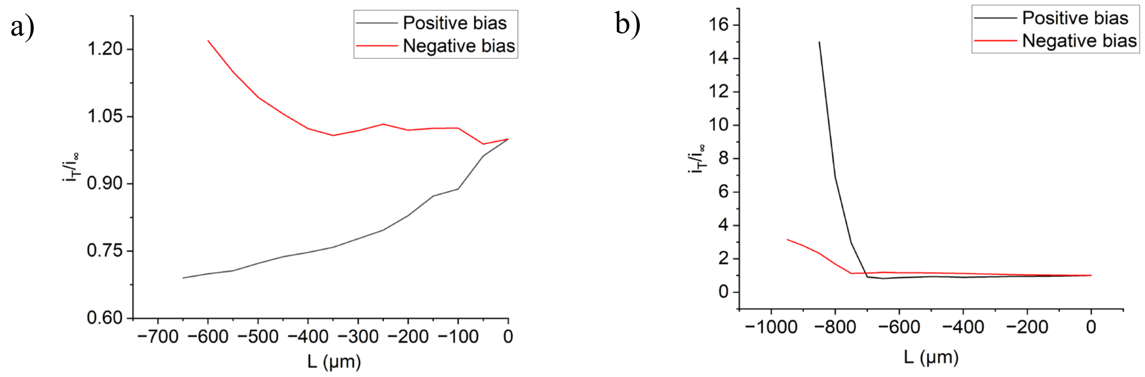

Figure S3: Approach curves above a catalyst-coated membrane at a) an intact site (negative feedback at positive bias and positive feedback at negative bias) and b) a perforated site (positive feedback at both biases).

The stability of the donor solution was assessed over a period of 5 hours by recording cyclic voltammograms (CV) every hour. Measurements were carried out using a voltammetric system (797 VA Computrace, Metrohm). A platinum microelectrode (6.1204.190) was used as the working electrode, a glassy carbon electrode (6.1247.000) as the counter electrode, and an Ag/AgCl electrode (6.0728.120, 3 M KCl, all from Metrohm) as the reference electrode.

Prior to the measurements, the solution was purged with nitrogen (1 bar) for 5 minutes to remove dissolved oxygen. Two CVs were then recorded. The exclusion of oxygen reflects the conditions during the DiffC-DC-SECM experiments, in which the donor solution is stored in a sealed syringe and continuously delivered to the donor reservoir via a syringe pump, thereby preventing exposure to air during the measurement.

The potential range was set from –0.30 V to +0.65 V with a sweep rate of 0.025 V s^{-1}. Figure S4 displays the second CV recorded at each time point. The data demonstrate a high electrochemical stability of the electrolyte over a period of at least 5 hours, covering the entire duration of the two area scans.

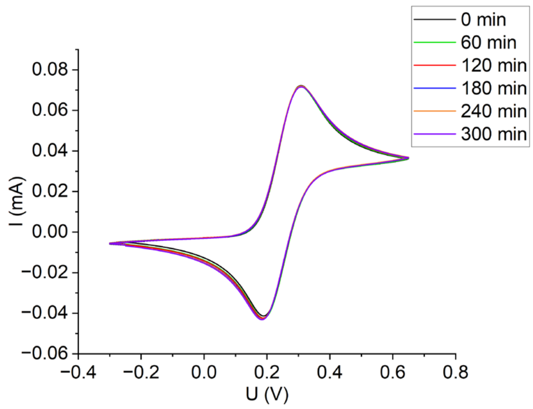

Figure S4: Cyclic voltammograms of the donor solution recorded over a period of 5 hours.

OCV AST Procedure

Prior to the aging test, the MEA was conditioned and a break-in procedure was carried out. The cell was first purged for several hours at 70 °C with humidified nitrogen (100 % RH), after which a break-in procedure was initiated. This procedure consisted of a one-hour repeating

cycle in which three potential levels (open-circuit voltage (OCV), 0.6 V, and 0.3 V) were each held for one minute under H_2/air atmosphere. Subsequently, a polarization curve was recorded. This cycle was repeated until two consecutive polarization curves showed no further deviation. The OCV test proposed by the U.S. Department of Energy (DoE) [2] to evaluate chemical membrane degradation was slightly modified for use with the single-cell test stand. All electrochemical measurements and the accelerated stress tests (AST) were conducted using a commercial 850e Fuel Cell Test System (Scribner Associates, Inc.), equipped with an 881 Frequency Response Analyzer, two potentiostats (850e and 885), and a PEM fuel cell fixture. The membrane electrode assembly (MEA) under investigation was a 25 cm^2 GORE® PRIMEA® Type A510.1/M765.08/C586.4 with 15.5 µm ePTFE membrane thickness (W. L. Gore & Associates), combined with H23C8 gas diffusion layers (GDL) with one-sided microporous coating (Freudenberg SE). The cell was held at OCV for 24 hours, with measurements recorded every 20 seconds (Figure S5). In contrast to the DOE specifications, the test was carried out with 1.5 bara on the cathode and 2 bara on the anode. Since a previous conducted AST revealed pronounced membrane damage only caused by a pressure differential, the present test was conducted with a pressure difference of 0.5 bar between anode and cathode. Subsequently, the recovery protocol was carried out, with the gas flow configuration summarized in Table S1. This process aimed to restore reversible voltage losses.

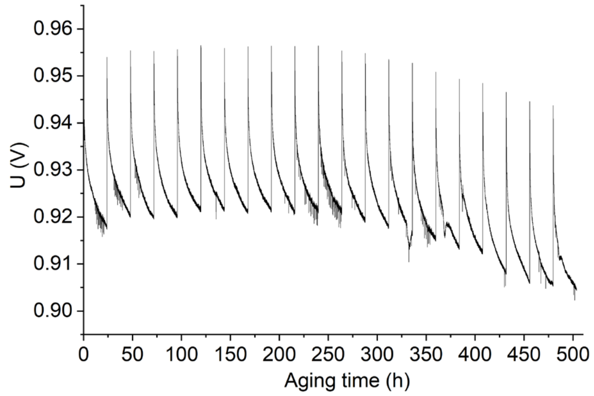

Figure S5: Illustration of the recorded fuel cell open-circuit voltage over the complete duration of the OCV AST including the recovery protocol.

Table S1: Gas flow configuration for the recovery protocol.

Step	Anode	Anode Flow (SLPM)	Cathode	Cathode Flow (SLPM)	Duration (s)
1	100 % N_2	2	100 % N_2	4	120
2	-	0	Air	4	900
3	100 % N_2	2	100 % N_2	4	120
4	100 % H_2	2	-	0	600
5	100 % H_2	2	Air	4	5

After the recovery, an analysis cycle was conducted, before the aging cycle was restarted. The DoE protocol recommends performing recovery and analysis at 80 °C with 100 % relative humidity (RH). However, due to the test stand's slow response to temperature and humidity changes, these steps were conducted under the same conditions as the OCV hold (90 °C, 30 % RH) to prevent delays from influencing the results. Following the AST, the GDLs were carefully detached using tweezers in preparation for the scanning electrochemical microscopy measurements.

During the analysis cycle, polarization curves were recorded following a procedure recommended by the DoE.[2] Prior to the measurement, a conditioning step was carried out in which the current density was held constant at 0.6 A cm^{-2} (corresponding to 15 A) for 20 minutes. Subsequently, 29 data points were recorded over a current density range from 0.02 A cm^{-2} to 2.4 A cm^{-2}. At each point, the current density was maintained for 3 minutes, and the cell voltage was recorded as the average value over the final 10 seconds of each interval. In deviation from the DoE protocol, these measurements were performed at a cell temperature of 90 °C and 30 % RH as mentioned before. Figure S6 displays the voltage and power density curves at the beginning and end of the AST over the course of 500 hours. The maximum power density decreased from 570 mW cm^{-2} to 537 mW cm^{-2}. This decline in both performance and polarization behavior indicates effective aging of the fuel cell system.

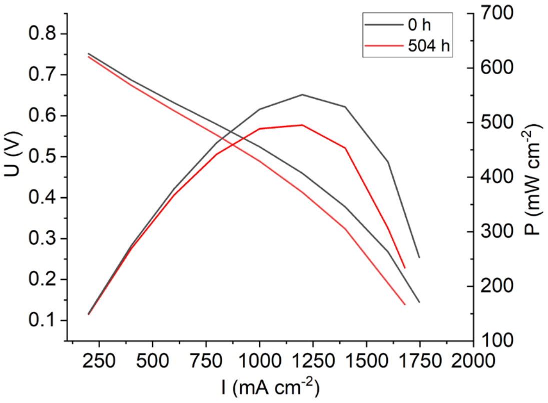

Figure S6: Fuel Cell polarization curve at the beginning and end of the OCV AST.

Linear sweep voltammetry (LSV) was used to monitor the development of hydrogen crossover through the membrane. A flow rate of 0.2 L min^{-1} of hydrogen was applied to the anode side and 3.0 L min^{-1} of nitrogen to the cathode side. The potential was swept from 0.05 V to 0.5 V versus the reference electrode at a scan rate of 1 mV s^{-1}. The hydrogen crossover current density increased from 6.28 mA cm^{-2} at the beginning of the test to 12.33 mA cm^{-2} to its end. This increase is attributed to elevated hydrogen permeability, likely resulting from the formation of structural defects such as cracks, pinholes, or membrane thinning.[3] According to the DoE, a hydrogen crossover current density of 15 mA cm^{-2} represents the threshold defining end-of-life for membrane materials in PEM fuel cells.[2]

[1] Bio-Logic Science Instruments Determining the Probe Diameter and RG Ratio in an SECM Experiment; 2016.
[2] Abdel-Baset, T.; Benjamin, T.; Borup, R.; et al. Fuel Cell Technical Team Roadmap Driving Research and Innovation for Vehicle Efficiency and Energy Sustainability Partnership 2017.
[3] Chandesris, M.; Vincent, R.; Guetaz, L.; Roch, J.-S.; Thoby, D.; Quinaud, M. Membrane degradation in PEM fuel cells: From experimental results to semi-empirical degradation laws. International Journal of Hydrogen Energy 2017, 42, 8139-8149.
